# Supplementary material for: Chromothripsis during telomere crisis is independent of NHEJ, and consistent with a replicative origin
Source: Genome Res. 2019 May;29(5):737–49. doi: 10.1101/gr.240705.118 (PMC6499312; doi:10.1101/gr.240705.118)
Supplement: Supplemental Material [file supp_gr.240705.118_Supplemental_file_1.zip › contigs/annotated_contigs/DB73/contig.2.DB73_length_269_mean_cov_4.843866171.docx]

**DB73_length_269_mean_cov_4.843866171**

```
TTCTTTC|TTTTTTTTTTCTAAGGCAGGGTCTCACTCTCTTGCCCAGACTGGAGTGCAGTGGCACGATCTCGGCTCACTCTGCCTCCCA
        >chr17:67381446-67381654 - E=3e-108
GGCTCAACCTCTGCTTCCCAGGCTCAAGTGATTCTCCTGCCTTGGCCTCCTGAGTAGCTGGGATTACAGGTTCACGCCACTACCCCCTC

GCTAATTTTTGTATTTTTAGTAGAGATGGGGTTTCACC|CCAATA|TATATATATATATATATATATATATATTGGACCAA|TATATAT
                                             >chrX:85100494-85100529 + E=1e-06
ATTTGG
```
